# Warning about Fatty Acid Compositions in Some Iranian Mayonnaise Salad Dressings

**Published:** 2010

**Authors:** Bahar Nazari, Sedigheh Asgary, Nizal Sarrafzadegan

**Affiliations:** 1 Research Assistant, Department of Basic Sciences, Isfahan Cardiovascular Research Center, Isfahan University of Medical Sciences,(IUMS), Isfahan, Iran; 2 Associate Professor of Pharmacognosy, Isfahan Cardiovascular Research Center, Applied Physiology Research Center, IUMS, Isfahan, Iran; 3 Professor of Cardiology, Isfahan Cardiovascular Research Center, IUMS, Isfahan, Iran

**Keywords:** Trans fatty acids, Saturated fatty acid, Unsaturated fatty acids, Mayonnaise, Iran

## Abstract

**Objectives:**

The beneficial or detrimental effects of dietary fats on health and well-being largely depend on their fatty acid composition. The contribution of high intake of trans fatty acids (TFAs) and saturated fatty acids (SFAs) to the risk of coronary heart disease is well documented. In this study, the fatty acid composition of different samples of mayonnaise salad dressing produced in Iran wasanalyzed.

**Methods:**

Three most consumed samples from four different brands of Iranian mayonnaises were purchased. Total lipids were extracted with Folch technique. All samples were transformed into methyl ester and analysis of fatty acid methyl esters were performed by gas chromatography (GC) with 60 meters capillary columns and flame ionization detectors.

**Results:**

TFAs ranged from 0.6% to 3.5%,with linolelaidic acid (C18:2 9t, 12t) being the most common form of fatty acid in Iranian mayonnaises, which had high amounts of SFAs (from18.1% to 24.9%) and unsaturated fatty acids (68.4%to 74.4%).

**Conclusions:**

The most common type of fatty acids in Iranian tested mayonnaises were unsaturated ones followed by SFAs. Significant variations were found among the contents of TFAs in these products, which is related to their procedure. Improvement of the fat quality in this highly consumed food product might have a long-term impact on prevention of chronic diseases.

## INTRODUCTION

Non-communicable diseases (NCDs), as coronary heart disease (CHD), diabetes and cancer, are major health problems in industrialized countries.^1^ It is estimated that by the year 2020, more than 60% of deaths in the developing countries will be related to NCDs.^2^ The recent interest in the development of evidence-based nutrition suggestions is because of the link between food diet, especially dietary fat, and NCDs.^3^ The influence of fats on body health chiefly depends on their fatty acid ingredient.^4^ Cis unsaturated fatty acids have advantageous effects on health.^5^,^6^ In contrast, studies have been published on the negative effects of trans fatty acids (TFAs) and saturated fatty acids (SFAs) on health specially on CHD.^7^,^8^ TFAs raise the risk of CHD ten times more than SFAs, hence TFAs are more risky than SFAs.^8^ TFAs with at least one double bond in the trans configuration are produced during the industrial procedures such as partial hydrogenation of vegetable oils^9^, refining of edible oils and food frying.^10^ Plant oils (margarine), mayonnaise, products baked with plant oils, and ready-made glazes and icings are foods with high amounts of TFA.^11^ A large body of evidence showed that dietary intakes of TFAs increase the risk of CHD because of negative effects on different risk factors such as serum lipids, lipoproteins^12^,^13^ and apolipoproteins^14^, inflammatory markers^15^,^16^, endothelial function, sudden death^17^,^18^ and diabetes mellitus.^18^ Because of the various adverse effects of manufactured TFAs, governing bodies recommend limiting TFAs in the food products.^19^ For instance, in 2003, the Danish government stated that “industrially produced TFAs should be limited to 2% of the total amount of fat or oil in a food”.^20^ The level and the kind of oils used in their preparations are effective on the amounts of TFAs in food products. This study was undertaken to quantify the amounts of the fatty acids exist in several different commercial mayonnaises produced in Iran, with particular attention to their TFAs content.

## METHODS

Three most consumed samples from four different brands of Iranian mayonnaises were purchased. The samples were determined via asking from some selected supermarkets about the brands people usually buy. In addition, we inquired this question randomly from selected group of people. After identifying these samples, to receive a homogenous sample of each brand, 70 gram of each three samples was mixed completely with stirrer. Ten g of each sample was drawn three times and prepared for fatty acid analysis. Total lipids were extracted by Folch technique.^21^ Thirty ml of a chloroform-methanol (2:1, v/v) mixture were added to 10 g of sample, which was shaken in a 250 ml Erlenmeyer flask by a magnetic stirrer for 45 min. Then, the solid phase was re-extracted two times more with the same volume of extractant. The liquid phases were combined in a separator funnel. Fourteen ml of saturated sodium chloride in water and 0.2 g of NaClO_4_ were added, and the mixture was slowly shaken. Then, the chloroform phase was filtered, dried with sodium sulfate and filtered again. Finally, the extractant was dried in a N_2_ until getting the constant weight.^21^ Samples of the lipid extract were taken and the fatty acid components converted to their respective methyl esters. Methylesterification of samples was done by the BF_3_-MeOH method.^22^ To 20 mg of mayonnaise samples were added 2 ml of 0.5 mol/L NaOH-methanol solution, and the mixture was heated at 100°C for 7 min. After cooling, 3 ml of 14% BF_3_-MeOH reagent was added, and the vessel was sealed and heated at 100°C for 5 min. After cooling, 2 ml of hexane and 7 ml of saturated NaCl solution were added, followed by a thorough shaking. The resulting hexane layer was used as a sample solution for gas chromatography (GC). Two mg of internal standard was added as a chloroform solution before esterification, and the solvent was removed under nitrogen. Then, the fatty acid composition of foods was obtained with a Young Lin capillary GC equipped with flame ionization detector (FID) and with a 60 m long, 0.25 mm inside diameter and 20 µm film thicknesses capillary column (TR-CN100), through a comparison of the retention relative times with those of commercial standards. Conditions of work included injection temperature of 240°C, detector temperature (FID) of 250°C, initial temperature of 90°C, and initial time of 5 min; then, continued with 150°C for 10 min, 200°C for 15 min and final temperature was 240°C for 20 min. Helium was the carrying gas. A pressure of 20 psi and a split ratio of 20/1 were applied.

### Statistical Analysis

Statistical analyses were performed with SPSS statistical package (version15.0). The differences in concentrations of contents of various mayonnaises were determined by one-way ANOVA followed by the Tukey post-hoc test. P value was significant if it was less than 0.05.

## RESULTS

Saturated fatty acids of different mayonnaises in Iran are shown in Table 1. The SFA amounts of mayonnaises varied from 18.1% to 24.9%.

**Table 1:** Total fat and saturated fatty acid (SFA) contents (%) of different mayonnaises marketed in Iran

	Mayonnaise1 (n=3)	Mayonnaise 2 (n=3)	Mayonnaise 3 (n=3)	Mayonnaise 4 (n=3)
Total fat	59.3%^a^	55.7%^a^	49.2%^a^	63.1%^a^
C4:0	0.0±0.0	0.0±0.0	0.0±0.0	0.0±0.0
C8:0	0.02±0.004^a^	0.0±0.0^b^	0.0±0.0^b^	0.0±0.0^b^
C10:0	0.02±0.008^a^	0.0±0.0^b^	0.0±0.0^b^	0.0±0.0^b^
C12:0	0.2±0.12^a^	0.02±0.01^c^	0.1±0.06^ab^	0.04±0.002^ab^
C14:0	1.3±0.92^a^	0.1±0.05^b^	0.02±0.002^c^	0.1±0.06^b^
C16:0	15.4±2.23^a^	14.2±1.20^b^	18.0±2.22^c^	12.2±1.55^b^
C17:0	0.02±0.006^a^	0.02±0.007^a^	0.002±0.001^b^	0.02±0.01^a^
C18:0	7.9±0.95^ab^	5.6±0.88^a^	5.4±1.21^a^	7.8±1.67^b^
Total SFA	24.9±3.32^a^	19.9±4.56^b^	18.1±2.98^b^	20.2±2.77^a^

C4:0 (Butyric acid); C8:0 (Caprylic acid); C10:0 (Capric acid); C12:0 (Lauric acid); C14:0 (Myristic acid); C16:0 (Palmitic acid); C17:0 (Heptadecanoic acid); C18:0 (Stearic acid).

Values in the same row with different superscripts (a, b, c and d) were significantly different (P<0.05.). In this cross sectional study, the amounts are expressed by mean ± SD. Analysis of variance (ANOVA) was applied to analyze the data.

The most common form of SFAs in Iranian mayonnaises is palmitic acid (C16:0) which ranged from 12.2% to 18.0%. Measure of Butyric acid (C4:0) was 0% in all samples. Table 2 represents TFA contents of different mayonnaises. TFAs were in the range 0.6% to 3.5%. Linolelaidic acid (C18:2 9t, 12t) was the most common form of fatty acid in these products. Amount of palmitelaidic acid (C16:1 t), petroselaidic acid (C18:1 6t), transvaccenic acid (C18:1 11t) and trans 13- octadecenoic acid (C18:1 13t) were 0% in the tested samples. Elaidic acid (C18:1 9t) existed also in a low amounts (0-0.6%). Total Cis unsaturated fatty acids (Cismono and polyunsaturated fatty acids) content of different mayonnaises are presented in Table 3. The highest amount of fatty acids was related to unsaturated fatty acids which ranged from 68.4%to 74.4%. Oleic acid (C18:1 9c) and linoleic acid (C18:2) were the most common forms of fatty acid in these products. Conjugated linoleic acid (CLA) was 0% in all samples.

**Table 2:** Trans fatty acid (TFA) content (%) of different mayonnaises marketed in Iran

	Mayonnaise 1 (n=3)	Mayonnaise 2 (n=3)	Mayonnaise 3 (n=3)	Mayonnaise 4 (n=4)
C16:1 t	0.0±0.0	0.0±0.0	0.0±0.0	0.0±0.0
C18:1 6t	0.0±0.0	0.0±0.0	0.0±0.0	0.0±0.0
C18:1 9t	0.3±0.12^a^	0.0±0.0^b^	0.2±0.13^c^	0.6±0.09^d^
C18:1 11t	0.0±0.0	0.0±0.0	0.0±0.0	0.0±0.0
C18:1 13t	0.0±0.0	0.0±0.0	0.0±0.0	0.0±0.0
C18:2 9t,12t	3.2±0.51^a^	0.9±0.32^ab^	0.4±0.12^b^	1.4±0.66^c^
Total TFA	3.5±1.23^a^	0.9±0.11^b^	0.6±0.05^b^	2.0±0.42^a^

(t=trans)

C16:1 t (Palmitelaidic acid); C18:1 9t (Elaidic acid); C18:1 6t (Petroselaidic acid); C18:1 11t (Transvaccenic acid); C18:1 13t (Trans 13-octadecenoic acid); C18:2-9t 12t (Linolelaidic acid)

Values in the same row with different superscripts (a, b, c and d) were significantly different (P<0.05.). In this cross sectional study the amount expressed by mean ± SD. Analysis of variance (ANOVA) was applied to analyze the data.

**Table 3:** Cis unsaturated fatty acids contents (%) of different mayonnaises marketed in Iran

	Mayonnaise 1 (n=3)	Mayonnaise 2 (n=3)	Mayonnaise 3 (n=3)	Mayonnaie 4 (n=3)
C16:1	1.0±0.52^a^	0.5±0.06^b^	0.6±0.1^b^	1.2±0.33^c^
C18:1 9c	32.2±2.88^a^	40.6±3.25^a^	41.1±5.56^a^	32.9±2.98^a^
C18:2	30.8±6.65^a^	27.7±5.10^ab^	24.7±2.22^b^	35.0±3.68^a^
CL^a^	0.0±0.0	0.0±0.0	0.0±0.0	0.0±0.0
C18:3	4.4±1.10^a^	5.6±1.87^a^	2.5±0.98^b^	4.5±0.55^a^
Total cis-unsaturated fatty acids	68.4±8.56	74.4±2.12	68.9±4.78	73.6±9.12

(c=cis)

C16:1 (Palmitoleic acid); C18:1 9c (Oleic acid); C18:2 (Linoleic acid); C18:2-c9t 11c or C18:2, t10 c12 (Conjugated linoleic acid (CLA)); C18:3 (Linolenic acid). Values in the same row with different superscripts (a, b, c and d) were significantly different (P<0.05.). In this cross sectional study, the amounts are expressed by mean ± SD. Analysis of variance (ANOVA) was applied to analyze the data.

## DISCUSSION

This study revealed that the most prominent fatty acids in Iranian tested mayonnaises were unsaturated ones followed by SFAs, with arange of TFAs from 0.6% to 3.5%. Although several studies have shown multiple adverse effects of TFAs on human health, we did not find no data about total fatty acid compositions, particularly TFAs, in Iranian products such as mayonnaises. This is especially important in Iran due to their extensive consumption by general population.

A previous study by Satchithanandam et al. showed that TFAs in the tested mayonnaises in the United States were from 0.0 to 2.2%.^23^ The effects of dietary fats on health are mainly depended on their fatty acid contents. Adverse effects of TFAs may be stronger for trans isomers of linoleic acid (*trans*-C18:2) and elaidic acid (*trans*-C18:1), rather than of palmitelaidic acid (*trans*-C16:1).^16^ Unfortunately, it should be mentioned that most of the TFAs found in these foods contain trans linoleic acid (*trans*-C18:2) isomers. It is documented that lauric acid (C12:0), myristic acid (C14:0) and palmitic acid (C16:0), decrease the activity of the LDL receptors, decrease the cellular LDL uptake^24^ and significantly increase total and LDL-cholesterol.^5^,^25^ Unfortunately, our samples contained high amounts of palmitic acid (16:0). A prospective cohort study demonstrated that foods such as mayonnaise, pizza/pies and canned fish could increase the risk of breast cancer in postmenopausal women.^26^ Also, using condiments and sauces is increasing in Iranian population and it is important to control their components. Our findings indicated significant variations among the contents of trans fats in Iranian commercially available mayonnaises, which was related to their procedure. The use of TFAs in Iranian food products should be limited as soon as possible. Food and Drug Administration (FDA) recommended that the amount of TFAs should be shown in the nutrition label of foods on a separate line immediately under the line of declaration of saturated fatty acids.^27^

In this study, our budget limitation did not allow to accurately determine all fatty acids (e.g., myristelaidoyl acid trans-C14:1) in the tested mayonnaises. Unfortunately, we were not able to analyze all different mayonnaises to report their fatty acid compositions separately. Future studies should take this point into account. Future clinical studies must be performed on estimating the dietary intakes of trans fats among Iranian population and determining their possible effects on human health.
